# A phase II study of docetaxel plus lycopene in metastatic castrate resistant prostate cancer

**DOI:** 10.1016/j.biopha.2021.112226

**Published:** 2021-09-24

**Authors:** Eric Zhuang, Edward Uchio, Michael Lilly, Xiaolin Zi, John P. Fruehauf

**Affiliations:** a Department of Medicine, Division of Hematology/Oncology, Chao Family Comprehensive Cancer Center, University of California Irvine, USA; b Department of Urology, University of California Irvine, USA

**Keywords:** Prostate cancer, Docetaxel, Lycopene, Phase II

## Abstract

We carried out a phase II study to investigate the activity of docetaxel plus lycopene in advanced castrate resistant adenocarcinoma of the prostate. Patients were chemotherapy and biological therapy naive. Docetaxel 75 mg/m^2^ was given every 21 days with daily oral lycopene 30 mg. The primary endpoint was a ≥50% reduction in PSA. Secondary endpoints were median time to PSA progression, duration of response and overall survival. Thirteen patients were initiated on protocol therapy. Median age was 77 (range 55–90). Twelve patients (92%) had bone metastases. Four patients (30%) had both bone and visceral metastases. PSA response was seen in 10 patients (76.9% [95% confidence interval (CI), 46.2–94.9%]). Two patients had stable disease (SD), yielding a disease control rate of 92%. Median time to PSA progression was 8 months [95% CI, 3.5–8.7]. Median duration of response (DOR) was 7.3 months [95% CI, 4.8–13.2]. Median overall survival at 5 years was 35.1 months [95% CI 25.7–57.7]. No new safety signals were noted. No patients experienced grade 3 or above anemia. One patient (7%) experienced febrile neutropenia. A PSA response rate of 76.9% and median survival of 35.1 months compares favorably to the 45% PSA response rate and 17.4 months median survival reported for the TAX 237 trialists. While our study was limited due to small sample size, our results suggest that the combination of docetaxel and lycopene merits further study.

## 1. Introduction

Prostate cancer is the most common malignancy among men and the second leading cause of cancer death in men in the United States. Among men in the U.S., the current estimated lifetime risk is about one in nine [1]. According to the SEER database, it is estimated that there will be about 248,530 new cases of prostate cancer in the U.S. in 2021 and about 34,130 deaths [1]. Treatment options for localized prostate cancer confined to the gland include active surveillance, radical prostatectomy, and radiation therapy [2,3]. While many cases of localized prostate cancer can be cured, one of three patients experience disease recurrence [4].

Metastatic prostate cancer presents a therapeutic challenge and is currently incurable. Most advanced prostate cancers initially respond well to androgen deprivation therapy (ADT) with luteinizing hormone releasing hormone (LHRH) analogues, orchiectomy, and/or testosterone receptor antagonists. After a variable time, however, all patients progress to castration resistant prostate cancer (CRPC), which has a median overall survival of approximately 2–2.5 years [5–7]. CRPC does not imply the disease is totally independent of androgens and second-generation anti-androgen therapy such as enzalutamide, apalutamide, or darolutamide may improve survival in certain groups. Unfortunately, in a majority of patients, disease progression will continue to occur despite ongoing anti-androgen therapy and they may require systemic treatment with cytotoxic agents such as docetaxel and cabazataxel.

Since 2004, docetaxel has become the standard first-line chemotherapy for the treatment of CRPC. Docetaxel is a taxoid derived from the needles of the European yew tree, Taxus baccata. In the TAX-327 trial, 1006 men with metastatic chemotherapy-naive CRPC were randomly assigned to receive either docetaxel 75 mg/m^2^ every three weeks, docetaxel 30 mg/m^2^ weekly, or mitoxantrone 12 mg/m^2^ every three weeks [8]. All patients also received prednisone 5 mg twice daily and were continued on maintenance gonadal androgen suppression if already on therapy. Long term follow-up of the trial demonstrated benefit in the every three week docetaxel group, with a median overall survival of 19.2 months versus 17.8 and 16.3 months for the weekly docetaxel and mitoxantrone regimens, respectively. Subgroup analyses also demonstrated the survival benefit in those older or younger than 65 years of age, those with and without pain at baseline, and those whose baseline prostate specific antigen (PSA) was greater than or less than the median of 116 ng/ml. Based on these results, docetaxel 75 mg/m^2^ plus prednisone 5 mg twice daily became the standard first line chemotherapy regimen for CRPC.

The incremental benefit that was observed in this trial carried with it the increased risk of toxicity. In addition, the optimal timing of initiating cytotoxic chemotherapy remains unclear as these trials included mixed populations of asymptomatic and symptomatic patients, rapidly progressing and not rapidly progressing patients. Hence, the asymptomatic and/or relatively stable CRPC patient provides a good opportunity to explore alternative agents in combination with docetaxel that may offer increased clinical benefit and less toxicity. Overall, improved understanding of the underlying mechanisms of progression of prostate cancer will hopefully lead to new treatment modalities.

Lycopene, a carotenoid which is responsible for the red pigment in tomatoes, has been suggested as a promising nutritional component in the chemoprevention of prostate cancer [9]. A large-scale study by Giovannucci et al. investigated 47,365 men and their dietary habits over 12 years [10]. They reported that patients with elevated lycopene intake had reduced risk of prostate cancer with a RR of 0.94 (*P <*0.05). Patients with high intake of tomato sauce, the primary source of bioavailable lycopene, was associated with even greater reduction in the risk of prostate cancer with a RR of 0.77 (*P <* 0.001).

Lycopene has been shown to accumulate in prostate tissue and function as a potent antioxidant in vitro [11–13]. The In vitro effects of lycopene on prostate cancer cells have been examined and have shown promising effects. A study of 41 patients with prostate cancer examined the in vitro effect of lycopene on prostate cancer specimens taken after radical prostatectomy and LnCap cells [14]. They observed that DNA synthesis was inhibited in a dose-dependent manner in both the primary prostate cancer specimens and in the LnCap cell line.

In terms of lycopene activity in CRPC, Ansari et al. reported positive findings in a prospective study of men with CRPC who received lycopene 10 mg per day [15]. Of the 20 men with metastatic CRPC, one patient experienced complete response (5%), six patients had partial response (30%), and ten had stable disease (50%). Schwenke et al. performed an open label phase 2 trial of 18 men who received lycopene 15 mg twice daily and showed stable disease in 29% of patients [16]. Schroder et al. reported a significant increase in free PSA-doubling time (1150 vs 445 days) in the treatment protocol group compared to controls, *P* = 0.041 [17]. This was a crossover study of patients treated with lycopene 15 mg and compounds for 10 weeks with a 4-week washout period. Lycopene was well tolerated overall, with minimal adverse events, and no grade 3 or higher adverse events reported in these studies.

The combination of lycopene with Docetaxel has also been examined. We previously compared lycopene alone, docetaxel alone and lycopene plus docetaxel in vitro cell cultures and in vivo with a CRPC xenograft model [18]. Given together, lycopene was shown to enhance the antitumor efficacy of docetaxel by approximately 38% when compared to docetaxel alone, *P* = 0.047. Mechanistically, lycopene was found to inhibit IGF-I signaling by down-regulation of IGF-I expression and upregulation of IGF-BPs, which in turn inhibits survivin. Survivin is an antiapoptotic protein and plays a role in cell cycle regulation. Based on these data, we carried out a phase II study examining the impact of docetaxel plus lycopene on subjects with CRPC who were candidates for first line docetaxel.

## 2. Methods

### 2.1. Patients

Eligible patients had histologically confirmed adenocarcinoma of the prostate and 2 rising pre-study PSA values ≥ 1 ng/ml at least one week apart within 28 days prior to enrollment. Patients had to have been unresponsive to ADT, as indicated by a rising PSA level above the ADT nadir, and had been surgically or medically castrated. If the patient already was being treated with medical castration, then the patient was continued on this treatment during the duration of the study. At least 28 days had to have elapsed since the completion of radiation therapy (maximum field less than 30% of the bone marrow) and the patient had to have recovered from side effects. At least 21 days had to have elapsed since surgery and the patient had to have recovered from side effects. Patients had to have no prior treatment with chemotherapy, biological therapy, or any other investigational drug for any reason within 28 days prior to the start of therapy and had recovered from toxicities of prior therapy to grade 1 or less.

Eligible patients had an ECOG performance of at least 0–2, had adequate hepatic function defined by: 1) normal serum bilirubin, 2) SGOT or SGPT ≤1.5 x the institutional upper limit of normal obtained within 14 days prior to start of therapy. Patients had adequate renal function as defined by serum creatinine ≤1.5 x the institutional upper limit of normal within 14 days prior to start of therapy. Hematological criteria included: 1) absolute neutrophil count >1500/mcl, 2) hemoglobin of>8.0 mg/dL, 3) white blood cell count >2500/mcl, 4) platelet count >100,000/mcl.

Exclusion criteria for this study included: 1) uncontrolled brain or spinal cord metastases, 2) history of congestive heart failure or myocardial infarction within the previous six months, 3) history of allergy or hypersensitivity to any component of the study drugs, 4) evidence of or history of bleeding diathesis or coagulopathy, 5) presence of chronic diarrhea, short bowel syndrome, pancreatic insufficiency or malabsorption, 6) presence of any severe medical condition, 7) concurrent use of any vitamin, herb, mineral supplement at least 14 days prior to start of therapy.

Clinical history was obtained along with physical examination and computed tomography, and bone scan were performed within 14 days of beginning treatment. Blood tests including measurement of serum PSA, testosterone, CBC + differential, Chem 12 panel were performed. All patients were provided with written informed consent. The study was approved by all institutional review boards in accordance with international standards of good clinical practice. The study was registered at ClinicalTrials.gov (NCT01882985).

### 2.2. Study treatment

All patients initially received docetaxel 75 mg/m^2^ every 21 days in combination with lycopene 30 mg orally once daily. Docetaxel was given as a 1-hour intravenous infusion on day 2 of a 21-day cycle, and lycopene 30 mg was taken once daily. Pre-medication was required with dexamethasone. 4 mg was given on day 1 with each cycle (before receiving docetaxel) to minimize side effects. 4 mg was given on day 2 (the day of receiving docetaxel) and with each cycle, and 4 mg was given on day 3 (the day after receiving docetaxel).

Treatment was planned to continue to a minmum of 4 cycles to reach the primary assessment point. Subjects were able to choose to continue treatment beyond this point until disease progression. Subjects already on ADT were continued on this therapy unchanged throughout the trial. Anti-inflammatory or narcotic analgesics and antiemetics were offered as needed. Packed red blood cell and platelet transfusions were administered as clinically indicated. Prophylactic use of hematopoietic growth factors to support neutrophil, red blood cell, or platelet counts were permitted during this study at the discretion of the treating physician. Pegfilgrastim rather than dose reduction was strongly considered if the patient experienced an episode of febrile neutropenia, or if neutropenia was prolonged (>7 days of neutropenia).

### 2.3. Study assessment

Medical history, physical examination, and blood test were repeated at 3–4 week intervals or every cycle. Imaging studies were performed at intervals of 12–15 weeks or every 4–5 cycles.

The primary endpoint was PSA response. A PSA response was defined as the proportion of subjects achieving a ≥50% reduction in PSA at any point after starting treatment. PSA responses were confirmed by a repeat assay in 3–4 weeks. The baseline PSA was at least 2 ng/ml to allow for evaluation for PSA response.

Secondary endpoints were median time to PSA progression, duration of response and overall survival. PSA progression was defined as an increase of greater than 25% in PSA (with a minimum increase of 2 ng/ ml) from pretreatment baseline value (if there was no PSA decrease during treatment) or from treatment nadir value. Time to PSA progression was defined as the time between first and last evaluation at which response criteria were met.

Adverse events were classified according to the Common Toxicity Criteria of the National Cancer Institute. Treatment was stopped for any of the following reasons: progression of disease, severe adverse event, death or withdrawal of consent.

### 2.4. Statistical analysis

PSA response rates and 95% confidence intervals were estimated using the Clopper-Pearson method. With a sample including 13 subjects, a two-sided 95% exact Clopper-Pearson confidence interval will extend ±0.21 from the observed proportion achieving PSA response. When the observed proportion is 0.63, a 95% confidence interval will extend from 0.42 to 0.84. With the proposed sample size and design, the true response rate will be ≥ 0.43 with probability 95% when the estimated response rate is greater than or equal to 0.63. Time to PSA progression, duration of response and overall survival were analyzed by means of the Kaplan-Meier method.

## 3. Results

### 3.1. Patients

Fourteen patients were screened and thirteen patients were enrolled onto study protocol. The baseline characteristics of the patients are summarized in Table 1. The median age was 77 years, with a range of 57–90 years. About 69% of the patients had an ECOG performance status of 0, and about 31% of the patients had an ECOG performance status of 1. Six patients (46%) had Gleason scores less than 7, four patients (31%) had Gleason scores greater than or equal to 8, and three patients had unavailable scores. Five patients (38%) had baseline serum PSA around the median value, while eight patients (62%) had baseline serum PSA elevated to greater than 20 ng/ml. Eleven patients (85%) had normal baseline serum alkaline phosphatase while two patients (15%) had elevated baseline alkaline phosphatase.

Most of the patients received the prescribed schedule and doses of docetaxel 75 mg/m^2^. Two patients (15%) required dose reduction to 60 mg/m^2^ due to adverse effects. One patient (7%) required one chemotherapy infusion to be delayed due a hospital admission. Treatment was discontinued in nine patients (78%) due to progression, in two patients (15%) due to death, in one patient (7%) due to loss of follow-up, and in one patient (7%) due to withdrawal of consent.

### 3.2. Clinical activity

The PSA response rate was 76.9% [95% CI, 46.2–94.9%], as assessed by the proportion of subjects who achieved ≥50% reduction in PSA after start of treatment, which consisted of ten of thirteen patients achieving PSA response (Table 2). Two patients had a best response of stable disease (SD), as defined by stable PSA levels, yielding an overall disease control rate (DCR) of 92.3% [95% CI, 63.9–99.8%]. One patient’s best overall response was progressive disease. This patient had a Gleason score of 10, serum PSA 1711.4 ng/ml, a serum alkaline phosphatase 2051 IU/L, both visceral and bony metastasis and ECOG 1 and died due to progression of disease one day after starting protocol therapy. The patient had presented to the infusion center the day prior, hemodynamically stable, at functional baseline.

**Fig. 1.**
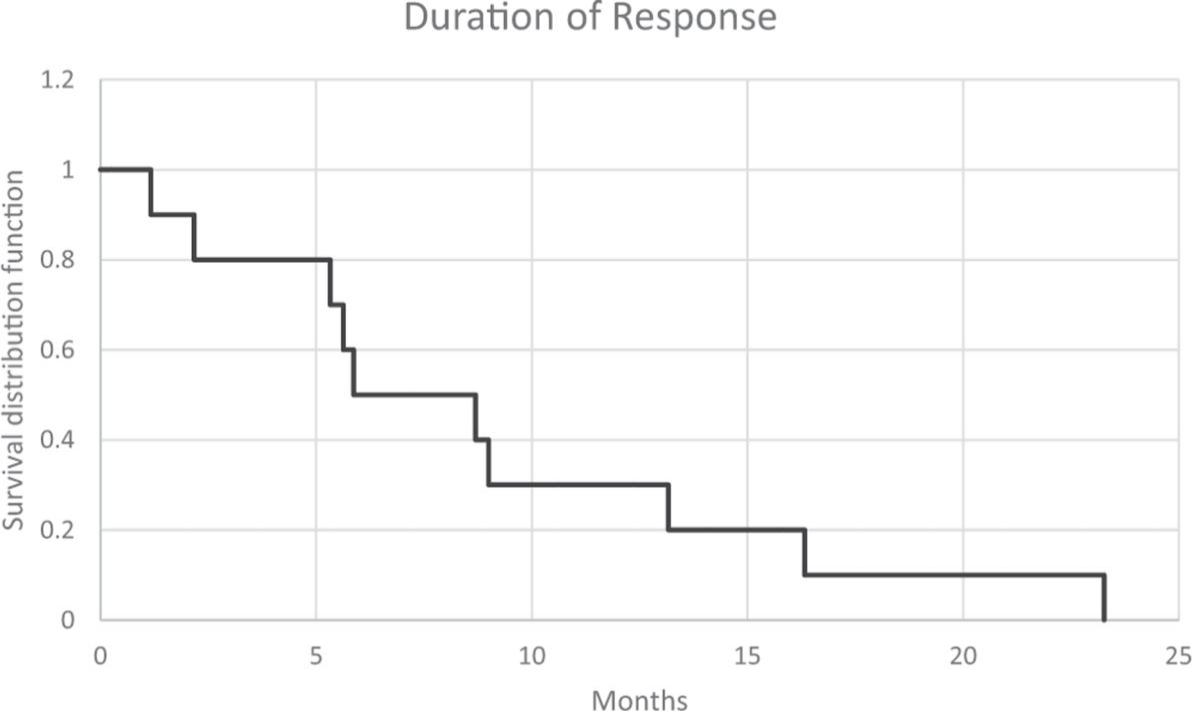
Kaplan-Meier plot for duration of response. Median 7.3 months [95% CI, 4.3 – 13.2].

The median duration of response was 7.3 months [95% CI, 4.8–13.2] (Fig. 1). Sub-group analysis of those with elevated baseline serum PSA ≥ 20 ng/ml had a median DOR 5.5 months [95% CI, 2.7–7.9] as compared to the group with *<* 20 ng/ml baseline serum which had median DOR 14.7 months [95% CI, 7.5–21.7].

**Fig. 2.**
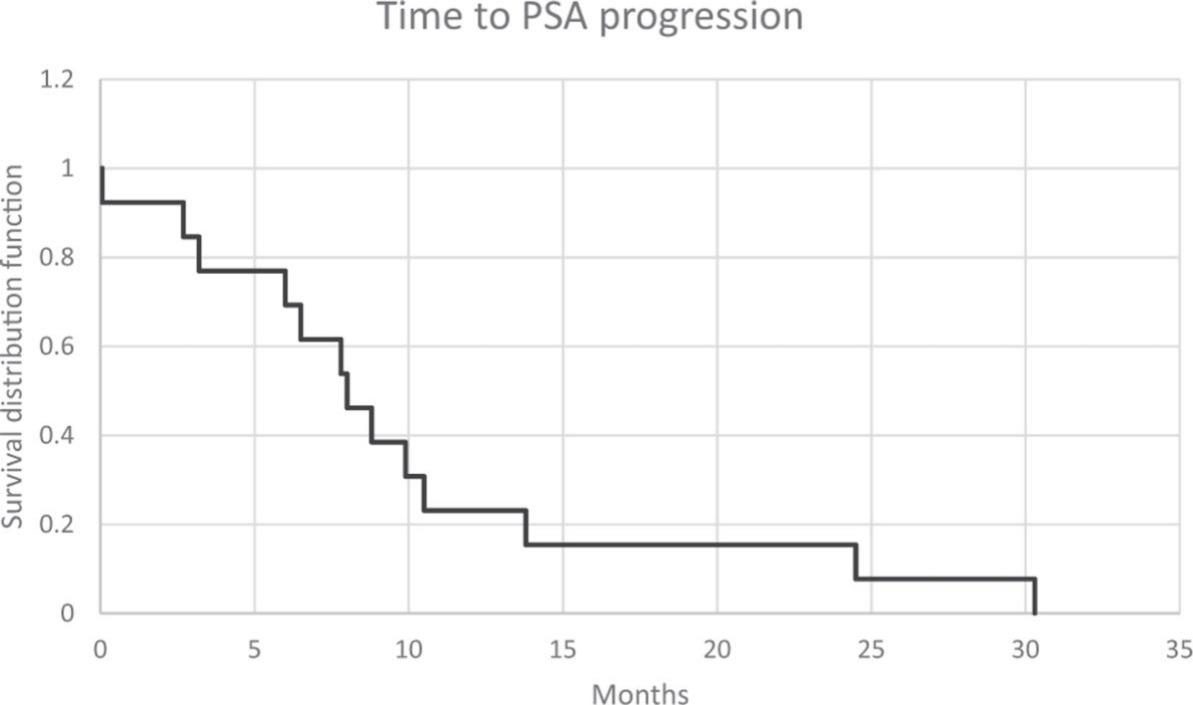
Kaplan-Meier plot of time to PSA progression. Median 8 months [95% CI, 5.5–14.7].

**Fig. 3.**
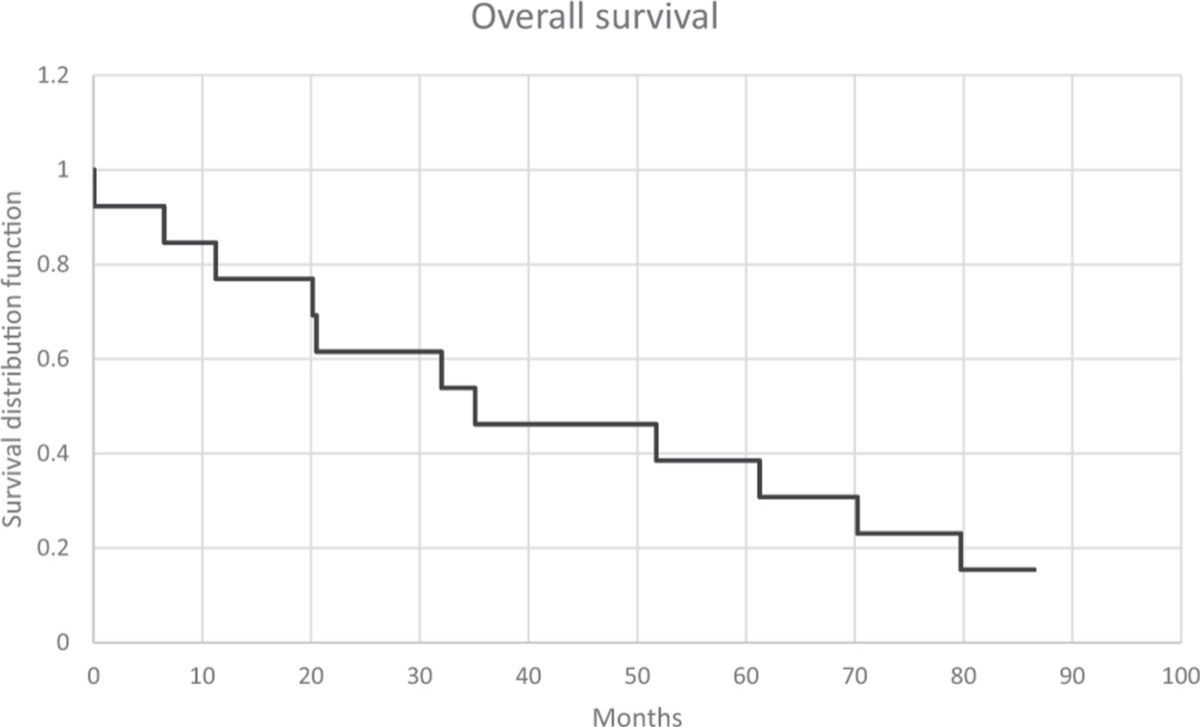
Kaplan-Meier plot of overall survival. Median 35.1 months [95% CI, 25.7–57.7].

Median time to PSA progression was 8 months [95% CI, 3.5–8.7]. Time to PSA progression was higher at 13.8 months [95% CI 7.5–21.7] in the sub group with baseline serum PSA *<* 20, while the group of patients with elevated baseline serum PSA ≥ 20 ng/ml showed median time to progression of 7 months [95% CI, 2.7–7.9]. On long term follow- up, median overall survival was 35.1 months [95% CI 25.7–57.7]. Kaplan-Meir survival curves for time to PSA progression and overall survival can be seen in Figs. 2 and 3.

### 3.3. Adverse events

Treatment with docetaxel plus lycopene was relatively well tolerated and no dose limiting toxicities were observed in the initial six patients enrolled in the study. Two patients (15%) required dose reduction and both were due to peripheral neuropathy. The most common adverse events (which were all low grade) occurring in at least 15% of patients included diarrhea, nausea or vomiting or both, peripheral neuropathy, weight loss, appetite changes, fatigue, onycholysis and alopecia neutropenia, and anemia, as listed in Table 3. Grade 3 or higher neutropenia occurred in four patients (31%). Only one other grade 3 or higher adverse events occurred; one patient experienced grade 3 peripheral neuropathy which required dose reduction as mentioned above. The incidence of febrile neutropenia was low, occurring in one patient only. Nine patients (69%) experienced anemia, however these were all low grade, and no grade 3 or above anemia occurred. No grade 5 toxicities were seen. There were no adverse events that led to discontinuation of treatment.

## 4. Discussion

In this phase 2 study, docetaxel plus lycopene was administered to patients with metastatic castrate resistant prostate cancer. For men with advanced prostate cancer, ADT is usually the first choice of treatment and can provide disease control for a substantial amount of time. However, the vast majority eventually progress on ADT to castrate resistant disease and may benefit from chemotherapy. Lycopene, a carotenoid found in tomatoes, is a compound found to have anti-prostate cancer activity and has been shown both in vitro and in vivo to enhance the activity of docetaxel [18]. The results of this clinical study demonstrated that docetaxel plus lycopene was highly active in metastatic CRPC, with a PSA response rate of 76.9%. Disease control rate was 92% when including patients who experienced stable disease on treatment. Although limited by a small number of patients and lack of a control arm, the efficacy observed in this study compares favorably with previously reported studies.

These results compare favorably to the landmark TAX 327 trial. The group given docetaxel every 3 weeks had a PSA response rate of 45%, and the group given docetaxel weekly had a PSA response rate of 48% [8]. The duration of response was similar between this trial and the group given docetaxel every 3 weeks (7.3 months versus 7.7 months, respectively). The characteristics of the patients in this study were similar to those seen in TAX 327. Most patients were older, chemotherapy-naive, had high baseline serum PSA, and bone metastases. Most had prior treatment with prostatectomy, radiotherapy and had two or more types of hormonal manipulations. Docetaxel plus lycopene had a slightly improved adverse event profile, with no grade 3 or 4 anemia or thrombocytopenia. It was overall better tolerated, with decreased rates of diarrhea (23% versus 32%), nausea or vomiting or both (30% versus 42%), alopecia (15% versus 65%), and stomatitis (7% versus 20%). No new toxicities were seen with docetaxel plus lycopene.

A phase 3 trial of 1168 patients with metastatic CRPC treated with cabazitaxel 20 mg/m^2^, cabazitaxel 25 mg/m2 or docetaxel 75 mg/m^2^ yielded similar findings. The authors found PSA response rates to be 60.7%, 68.7%, and 68.4%, respectively [19]. Median time to PSA progression was also similar to our study (8 months) compared to each study group (8.2 months, 9.2 months, 8.3 months, respectively). Rates of adverse events were consistent as well, though the cabazitaxel groups had lower rates of febrile neutropenia (2.4%) compared to our study.

Multiple large phase 3 trials have studied docetaxel in combination with other agents. One trial that evaluated docetaxel plus bevacizumab versus docetaxel alone in men with metastatic CRPC found that patients on the docetaxel plus bevacizumab arm had a PSA response rate of 69.5% and median PFS of 9.9 months [20]. However, this improvement in response and PFS was associated with significantly greater toxicities (grade 3 or greater adverse events 75.4% versus 56.2% in placebo). The VENICE trial studied docetaxel plus aflibercept, a recombinant human fusion protein that binds A and B isoforms of VEGF and placental growth factor [21]. This trial found no differences in response rates nor survival, with the cost of added toxicites. The ASCENT trial that examined docetaxel plus calcitriol was halted early as more deaths were noted in the calcitriol arm [22].

In conclusion, our findings suggest that docetaxel plus lycopene has favorable activity in patients with metastatic castrate resistant prostate cancer. Duration of response and time to PSA progression were comparable to previous trials. Treatment was relatively well tolerated, and adverse event rates were similar if not improved compared to previous trials. Lycopene alone has demonstrated single-agent activity towards prostate cancer and is very well tolerated with low risk of adverse events. In addition, lycopene may have synergistic activity with docetaxel through downregulation of IGF-I signaling inhibition and decreased survivin expression. Further study on docetaxel plus lycopene is warranted.

## Figures and Tables

**Table 1:** Patient characteristics.

	Docetaxel plus lycopene (*N* = 13)
**Median age, years**	77
**Range**	57–90
**ECOG performance status, n(%)**	
0	9 (69.2)
1	4 (30.8)
**Gleason score, n(%)**	
≤ 7	6 (46.2
8–10	4 (30.1)
Unavailable	3 (23.1)
**Prior treatment, n(%)**	
Prostatectomy	5 (38.5)
Hormone therapy	12 (92.3)
Radiotherapy	8 (61.5)
**Hormonal manipulations, n(%)**	
1	1 (7.6)
2	6 (46.2)
> 2	5 (38.5)
**Baseline serum PSA, n(%)**	
Median (ng/ml)	5 (38.5)
≥ 20 ng/ml (%)	8 (61.5)
**Baseline serum Alk Phos, n(%)**	
Normal	11 (84.6)
Elevated	2 (15.4)
**Extent of disease, n(%)**	
Bone metastases	12 (92.3)
Visceral metastases	4 (30.1)
Bone and visceral	4 (30.1)
**Subsequent treatment, n(%)**	
Chemotherapy	6 (46.1)
Hormonal therapy	8 (61.5)
Immunotherapy	3 (23.1)
Radiotherapy	2 (15.4)
PARP inhibitor therapy	2 (15.4)

* Because of round not all percentages total 100

**Table 2:** Treatment outcomes.

	Docetaxel plus lycopene (*N* = 13)
**PSA Response (≥50% reduction in serum PSA)**	
Events (n)	10
Rate (%)	76.9
95% CI	46.18–94.96%
**Disease control rate (%)**	92.3
95% CI	63.97–99.8%
**Duration of response (mo)**	
Median	7.3
95% CI	4.8–13.2
**Progression free survival (mo)**	
Median	8
95% CI	5.5–14.7
**Overall survival (mo)**	
Median	35.1
95% CI	25.8–57.8

**Table 3:** Adverse events.

	Total (%)	Grade ≥ 3 (%)
Neutropenia	8 (61)	4 (30)
Febrile neutropenia	1 (7)	1 (7)
Anemia	9 (69)	0
Diarrhea	3 (23)	0
Hematuria	1 (7)	0
Nausea, Vomiting, or Both	4 (30)	0
Peripheral neuropathy	6 (46)	1 (7)
Weight loss	3 (23)	0
Loss of appetite	2 (15)	0
Fatigue	8 (61)	0
Onycholysis	6 (46)	0
Macular rash	1 (7)	0
Hyponatremia	1 (7)	0
Alopecia	2 (15)	0
Mucositis or Stomatitis	1 (7)	0

## References

[1] Siegel RL, Miller KD, Jemal A, Cancer statistics, CA Cancer J. Clin. 71 (2021) 7–33. 33433946 10.3322/caac.21654

[2] Wilt TJ, Vo TN, Langsetmo L, Dahm P, Wheeler T, Aronson WJ, Cooperberg MR, Taylor BC, Brawer MK, Radical prostatectomy or observation for clinically localized prostate cancer: extended follow-up of the Prostate Cancer Intervention versus Observation Trial (PIVOT), Eur. Urol. 77 (6) (2020) 713–724. 32089359 10.1016/j.eururo.2020.02.009

[3] Hamdy FC, Donovan JL, Lane JA, Mason M, Metcalfe C, Holding P, Davis M, Peters TJ, Turner EL, Martin RM, Oxley J, Robinson M, Staffurth J, Walsh E, Bollina P, Catto J, Doble A, Doherty A, Gillatt D, Kockelbergh R, Kynaston H, Paul A, Powell P, Prescott S, Rosario DJ, Rowe E, Neal DE, ProtecT Study G, 10-year outcomes after monitoring, surgery, or radiotherapy for localized prostate cancer, N. Engl. J. Med 375 (15) (2016) 1415–1424. 27626136 10.1056/NEJMoa1606220

[4] Punnen S, Cooperberg MR, D’amico AV, Karakiewicz PI, Moul JW, Scher HI, Schlomm T, Freedland SJ, Management of biochemical recurrence after primary treatment of prostate cancer: a systematic review of the literature, Eur. Urol. 64 (6) (2013) 905–915. 23721958 10.1016/j.eururo.2013.05.025

[5] Wang L, Paller CJ, Hong H, De Felice A, Alexander GC, Brawley O, Comparison of systemic treatments for metastatic castration-sensitive prostate cancer: a systematic review and network meta-analysis, JAMA Oncol. 7 (3) (2021) 412–420. 33443584 10.1001/jamaoncol.2020.6973 PMC7809610

[6] Kessel A, Kohli M, Swami U, Current management of metastatic castration- sensitive prostate cancer, Cancer Treat. Res. Commun. 28 (2021), 100384. 10.1016/j.ctarc.2021.100384 33951556

[7] Halabi S, Kelly WK, Ma H, Zhou H, Solomon NC, Fizazi K, Tangen CM, Rosenthal M, Petrylak DP, Hussain M, Vogelzang NJ, Thompson IM, Chi KN, de Bono J, Armstrong AJ, Eisenberger MA, Fandi A, Li S, Araujo JC, Logothetis CJ, Quinn DI, Morris MJ, Higano CS, Tannock IF, Small EJ, Meta- analysis evaluating the impact of site of metastasis on overall survival in men with castration-resistant prostate cancer, J. Clin. Oncol. 34 (14) (2016) 1652–1659. 26951312 10.1200/JCO.2015.65.7270 PMC4872320

[8] Tannock IF, de Wit R, Berry WR, Horti J, Pluzanska A, Chi KN, Oudard S, Théodore C, James ND, Turesson I, Rosenthal MA, Eisenberger MA, Docetaxel plus prednisone or mitoxantrone plus prednisone for advanced prostate cancer (Oct.), N. Engl. J. Med. 351 (15) (2004) 1502–1512. 15470213 10.1056/NEJMoa040720

[9] Clinton SK, Lycopene: chemistry, biology, and implications for human health and disease (Feb.), Nutr. Rev. 56 (2) (1998) 35–51. 9529899 10.1111/j.1753-4887.1998.tb01691.x

[10] Giovannucci E, Rimm EB, Liu Y, Stampfer MJ, Willett WC, A prospective study of tomato products, lycopene, and prostate cancer risk (Mar.), J. Natl. Cancer Inst. 94 (5) (2002) 391–398. 11880478 10.1093/jnci/94.5.391

[11] Campbell JK, Engelmann NJ, Lila MA, Erdman JW Jr, Phytoene, phytofluene, and lycopene from tomato powder differentially accumulate in tissues of male fisher 344 rats (Dec.), Nutr. Res. 27 (12) (2007) 794–801. 19050742 10.1016/j.nutres.2007.09.015 PMC2330325

[12] Wertz K, Siler U, Goralczyk R, Lycopene: modes of action to promote prostate health (Oct.), Arch. Biochem. Biophys. 430 (1) (2004) 127–134. 15325920 10.1016/j.abb.2004.04.023

[13] Erdman JW Jr, Ford NA, Lindshield BL, Are the health attributes of lycopene related to its antioxidant function? (Mar.), Arch. Biochem. Biophys. 483 (2) (2009) 229–235. 18983972 10.1016/j.abb.2008.10.022 PMC2745920

[14] Barber NJ, Zhang X, Zhu G, Pramanik R, Barber JA, Martin FL, Morris JDH, Muir GH, Lycopene inhibits DNA synthesis in primary prostate epithelial cells in vitro and its administration is associated with a reduced prostate-specific antigen velocity in a phase II clinical study, Prostate Cancer Prostatic Dis. 9 (4) (2006) 407–413. 16983396 10.1038/sj.pcan.4500895

[15] Ansari MS, Gupta NP, Lycopene: a novel drug therapy in hormone refractory metastatic prostate cancer. (Oct.), Urol. Oncol. 22 (5) (2004) 415–420. 15464923 10.1016/j.urolonc.2004.05.009

[16] Schwenke C, Ubrig B, Thürmann P, Eggersmann C, Roth S, Lycopene for advanced hormone refractory prostate cancer: a prospective, open phase II pilot study (Mar.), J. Urol. 181 (3) (2009) 1098–1103. 19150092 10.1016/j.juro.2008.11.012

[17] Schröder FH, Roobol MJ, Boevé ER, de Mutsert R, Zuijdgeest-van Leeuwen SD, Kersten I, Wildhagen MF, van Helvoort A, Randomized, doubleblind, placebo-controlled crossover study in men with prostate cancer and rising PSA: effectiveness of a dietary supplement, Eur. Urol. 48 (2005) 922–930. 16263208 10.1016/j.eururo.2005.08.005

[18] Tang Y, Parmakhtiar B, Simoneau AR, Xie J, Fruehauf J, Lilly M, Zi X, Lycopene enhances docetaxel’s effect in castration-resistant prostate cancer associated with insulin-like growth factor I receptor levels, Neoplasia 13 (2) (2011) 108–119, 10.1593/neo.101092. 21403837 PMC3033590

[19] Oudard S, Fizazi K, Sengeløv L, Daugaard G, Saad F, Hansen S, Hjälm-Eriksson M, Jassem J, Thiery-Vuillemin A, Caffo O, Castellano D, Mainwaring PN, Bernard J, Shen L, Chadjaa M, Sartor O, Cabazitaxel versus docetaxel as first-line therapy for patients with metastatic castration-resistant prostate cancer: a randomized phase III trial-FIRSTANA (Oct.), J. Clin. Oncol. 35 (28) (2017) 3189–3197. 28753384 10.1200/JCO.2016.72.1068

[20] Kelly WK, Halabi S, Carducci M, George D, Mahoney JF, Stadler WM, Morris M, Kantoff P, Monk JP, Kaplan E, Vogelzang NJ, Small EJ, Randomized, double-blind, placebo-controlled phase III trial comparing docetaxel and prednisone with or without bevacizumab in men with metastatic castration- resistant prostate cancer: CALGB 90401, J. Clin. Oncol. 30 (13) (2012) 1534–1540, 10.1200/JCO.2011.39.4767. 22454414 PMC3383121

[21] Tannock IF, Fizazi K, Ivanov S, Karlsson CT, Fléchon A, Skoneczna I, Orlandi F, Gravis G, Matveev V, Bavbek S, Gil T, Viana L, Arén O, Karyakin O, Elliott T, Birtle A, Magherini E, Hatteville L, Petrylak D, Tombal B, Rosenthal M, VENICE i, Aflibercept versus placebo in combination with docetaxel and prednisone for treatment of men with metastatic castration-resistant prostate cancer (VENICE): a phase 3, double-blind randomised trial, Lancet Oncol. 14 (8) (2013) 760–768, 10.1016/S1470-2045(13)70184-0. 23742877

[22] Scher HI, Jia X, Chi K, de Wit R, Berry WR, Albers P, Henick B, Waterhouse D, Ruether DJ, Rosen PJ, Meluch AA, Nordquist LT, Venner PM, Heidenreich A, Chu L, Heller G, Randomized, open-label phase III trial of docetaxel plus high-dose calcitriol versus docetaxel plus prednisone for patients with castration-resistant prostate cancer, J. Clin. Oncol. 29 (16) (2011) 2191–2198, 10.1200/JCO.2010.32.8815. 21483004

